# Patient-derived xenografts as preclinical neuroblastoma models

**DOI:** 10.1007/s00441-017-2687-8

**Published:** 2017-09-19

**Authors:** Noémie Braekeveldt, Daniel Bexell

**Affiliations:** Translational Cancer Research, Department of Laboratory Medicine, Lund University, Medicon Village 404:C3, SE-223 81 Lund, Sweden

**Keywords:** Neuroblastoma, Pediatric cancer, Mouse model, Patient-derived xenograft (PDX)

## Abstract

The prognosis for children with high-risk neuroblastoma is often poor and survivors can suffer from severe side effects. Predictive preclinical models and novel therapeutic strategies for high-risk disease are therefore a clinical imperative. However, conventional cancer cell line-derived xenografts can deviate substantially from patient tumors in terms of their molecular and phenotypic features. Patient-derived xenografts (PDXs) recapitulate many biologically and clinically relevant features of human cancers. Importantly, PDXs can closely parallel clinical features and outcome and serve as excellent models for biomarker and preclinical drug development. Here, we review progress in and applications of neuroblastoma PDX models. Neuroblastoma orthotopic PDXs share the molecular characteristics, neuroblastoma markers, invasive properties and tumor stroma of aggressive patient tumors and retain spontaneous metastatic capacity to distant organs including bone marrow. The recent identification of genomic changes in relapsed neuroblastomas opens up opportunities to target treatment-resistant tumors in well-characterized neuroblastoma PDXs. We highlight and discuss the features and various sources of neuroblastoma PDXs, methodological considerations when establishing neuroblastoma PDXs, in vitro 3D models, current limitations of PDX models and their application to preclinical drug testing.

## Introduction

One of the main reasons for the high attrition rate in oncology drug development is a lack of preclinical models that recapitulate the genotype and phenotype of the patient’s disease. Xenografts based on conventional cancer cell lines have been used for decades and while this model system can provide valuable data, cultured cell lines that have adapted to the in vitro microenvironment often differ from the original tumor found in patients. Specifically, the addition of fetal calf serum to the culture medium can lead to cellular differentiation and significant genetic aberrations (Lee et al. 2006). Gene expression profiling has further demonstrated that cell lines obtained from diverse tumors resemble each other more than the corresponding clinical samples from which they were derived and serum-cultured cell lines can lose drug resistance mechanisms (Gillet et al. 2011). Although cell line-derived xenografts have contributed to the identification and testing of many classical cytotoxic drugs, these models tend to be less predictive of the action of targeted therapies (Johnson et al. 2001) and the US Food and Drug Administration approval rate for targeted therapies in oncology is as low as 5–7% (Sharpless and Depinho 2006). Given the huge financial and human costs associated with the numerous examples of clinical trial failure, there is a strong imperative to improve preclinical models in oncology.

Patient-derived xenografts (PDXs) are generated from the subcutaneous or orthotopic implantation of intact patient tumor fragments directly into immunodeficient mice or rats, thereby avoiding in vitro adaptation. The concept of PDXs has been around for decades but interest in PDXs has recently increased due to greater insights into the limitations of classical xenografts and the development of personalized cancer medicines based on genomic profiling. Thus, PDXs have been established and characterized for various malignant tumor types including breast cancer, malignant melanoma, colorectal cancer, pancreatic adenocarcinoma, non-small cell lung cancer and more (Hidalgo et al. 2014; Tentler et al. 2012). In these diverse tumors, PDXs can recapitulate the histopathological hallmarks, genetic pathways and mutational patterns of the corresponding patient tumors (Hidalgo et al. 2014; Tentler et al. 2012). Initial studies also indicate that the proteomic profiles of PDXs can resemble those of the corresponding patient tumors (Huang et al. 2017; Li et al. 2016). A series of well-characterized PDXs can thus cover the different molecular subsets of a specific tumor type (Hidalgo et al. 2014; Tentler et al. 2012). Once a PDX has been established, tumors can be serially passaged to next-generation recipients and these models generally retain their molecular features after serial passaging (Hidalgo et al. 2014; Tentler et al. 2012). Major applications of PDXs include drug testing/screening, biomarker discovery and exploration of treatment resistance. Several studies have shown that results derived from PDXs parallel clinical outcomes (Malaney et al. 2014; Rosfjord et al. 2014). Recently, a high-throughput screening program using 1000 PDXs with diverse driver mutations was used to demonstrate reproducible associations between genotype and drug responses, arguing for PDXs as an improved preclinical evaluation system compared to conventional cancer cell lines (Gao et al. 2015). In addition, PDXs have been used to examine drug resistance mechanisms and to identify therapeutic strategies to target drug resistance (Das Thakur et al. 2013; Girotti et al. 2015).

Neuroblastoma is the most common pediatric extracranial solid tumor. Although many neuroblastoma patients now survive, high-risk neuroblastoma is still potentially lethal. Furthermore, lifelong and severe side effects are common in survivors. Therefore, predictive preclinical models and novel treatment strategies are warranted for children with high-risk neuroblastoma. In this article, we review the features and various sources of neuroblastoma PDXs, 3D in vitro models, current limitations of PDX models, application of neuroblastoma PDXs to preclinical drug testing, methodological issues for neuroblastoma PDX establishment and future perspectives.

## Neuroblastoma

Neuroblastoma accounts for around 15% of pediatric oncology deaths. Most primary neuroblastomas arise within the abdomen, in particular from the adrenal gland, although tumors can arise anywhere along the sympathetic nervous system. Neuroblastomas are clinically diverse, ranging from spontaneously regressing to metastatic and treatment-resistant disease. Neuroblastoma patients are classified according to pretreatment risk groups: very low, low, intermediate and high risk. *MYCN* amplification or 11q loss of heterozygosity is found in many high-risk neuroblastomas and correlates with poor prognosis and treatment relapse (Cohn et al. 2009). Other common chromosomal copy number changes that associate with aggressive disease include 1p deletion and 17q gain. Metastases to bone, bone marrow and liver are frequent in high-risk patients. Treatment strategies for high-risk disease include high-dose chemotherapy, surgery, radiotherapy and anti-GD2 immunotherapy (Maris et al. 2007).

Genome-wide exome sequencing studies have revealed a relative paucity of recurrent somatic mutations at diagnosis (Eleveld et al. 2015; Pugh et al. 2013). However, relapsed neuroblastomas contain higher numbers of recurrent and targetable mutations such as those in *ALK* (Eleveld et al. 2015; Padovan-Merhar et al. 2016; Schleiermacher et al. 2014; Schramm et al. 2015). Importantly, mutations enriched in relapsed neuroblastomas are predicted to activate the RAS-MAPK pathway, YAP and/or epithelial–mesenchymal transition (EMT) (Eleveld et al. 2015; Padovan-Merhar et al. 2016; Schramm et al. 2015). Furthermore, high-risk neuroblastoma can be classified into different biological subsets based on molecular characterization including *MYCN* amplification status, c-MYC expression, *ALK* and *ATRX* mutations, *TERT* rearrangements and alternative mechanisms of telomere lengthening (ALT) (Peifer et al. 2015; Valentijn et al. 2015).

The recent molecular characterization of high-risk neuroblastoma has thus revealed that relapsed neuroblastomas contain genomic changes linked to targetable oncogenic pathways. These findings strengthen the case for molecular profiling of neuroblastomas pre- and post-treatment for personalized medicine strategies. With this in mind, well-characterized neuroblastoma PDXs covering the different molecular subsets could play a crucial role in preclinical drug testing of relapsed neuroblastoma.

## Features and sources of neuroblastoma PDXs

The National Cancer Institute (NCI) implemented the Pediatric Preclinical Testing Program (PPTP), which generated hundreds of pediatric subcutaneous tumor xenografts including neuroblastomas. Importantly, many of these xenografts were established at relapse following multi-modal chemotherapy. Several anti-cancer agents have been tested in the PPTP using in vivo and in vitro models and the effects of some of these agents are consistent with their known clinical activity (Houghton et al. 2007; Kang et al. 2011).

**Fig. 1:**
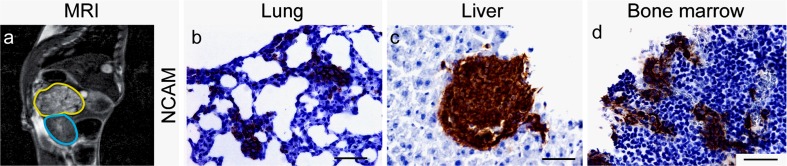
Neuroblastoma patient-derived orthotopic xenografts (PDOXs). **a**) Magnetic resonance imaging depicting a neuroblastoma PDOX (*circled in yellow*) located adjacent to the left kidney (*in blue*). Neuroblastoma PDOXs retain spontaneous metastatic capacity to distant organs including **b** lungs, **c** liver and **d** bone marrow, making them suitable models for studying and targeting human neuroblastoma metastasis. Metastatic cells are shown by expression of the neuroblastoma marker NCAM/CD56. *Scale bar* 50 μm

We have established neuroblastoma patient-derived orthotopic xenografts (PDOXs) through implantation of either fresh or viably cryopreserved naïve and relapsed high-risk neuroblastoma tumor fragments (Braekeveldt et al. 2015). The time taken for tumors to establish can vary significantly, in our hands from 2 to 10 months (Braekeveldt et al. 2015). These PDOXs retain the chromosomal copy number profile (1p del, *MYCN* amplification and 17q gain), neuroblastoma protein markers (synaptophysin, chromogranin A, neural cell adhesion molecule, and tyrosine hydroxylase), cellular differentiation status and proliferative index of their corresponding patient tumors. In contrast to many conventional cell-derived xenografts, PDOXs invade the surrounding tissues. Importantly, PDOXs spontaneously metastasize to the liver, lungs and bone marrow (Fig. 1), in contrast to many conventional cell line-derived neuroblastoma orthotopic xenografts, which usually only give rise to single metastatic cells (Braekeveldt et al. 2015; Khanna et al. 2002). Similarly, studies on many other tumor types have shown that PDOXs retain higher metastatic capacity than subcutaneous PDXs (Hoffman 2015). Thus, it seems that neuroblastoma PDOXs are a promising model for studying and targeting spontaneous human neuroblastoma metastases.

The Childhood Solid Tumor Network (CSTN) at St Jude Children’s Research Hospital has established and characterized a number of pediatric cancer PDXs including neuroblastoma PDOXs. These PDOXs have undergone comprehensive molecular characterization including genomic and epigenomic analyses as well as drug sensitivity testing using short-term cultured cells (Stewart et al. 2015, 2016). Notably, both gene expression and DNA methylation analyses revealed good correlations between the patient sample and the corresponding PDX (Stewart et al. 2015). Researchers at Institut Curie recently established and analyzed neuroblastoma PDXs models to decipher neuroblastoma heterogeneity (Boeva et al. 2017). The Children’s Oncology Group (COG) cell culture and xenograft repository (http://www.cogcell.org/xenografts.php), the Targeting Of Resistance in PEDiatric Oncology program (TORPEDO, http://www.transcanfp7.eu/index.php/abstract/torpedo.html), the IMI2 ITCC-P4 program (http://cordis.europa.eu/project/rcn/210764_en.html) and the recently established NCI-funded Pediatric Preclinical Testing Consortium (PPTC, (http://www.ncipptc.org/) are other programs that aim to establish and characterize pediatric PDXs. In addition, The EuroPDX consortium, although mainly focused on adult cancers, has started to include pediatric PDXs (http://www.europdx.eu/).

Neuroblastoma PDXs and PDOXs have thus been established and characterized in various laboratories (summarized in Table 1) and additional models are continuously being established. Many important molecular and phenotypic features of patient tumors are retained in PDXs. How serial in vivo passaging of neuroblastoma PDXs affects the genotype and phenotype is less well understood and needs to be determined, since serially passaged PDXs are often used in drug testing studies.

**Table 1:** Major sources of neuroblastoma PDX/PDOX models^a^

Organization	Website	References
Pediatric Preclinical Testing Program (PPTP)	http://gccri.uthscsa.edu/pptp/	Houghton et al. 2007
Lund University		Braekeveldt et al. 2015, 2016
Childhood Solid Tumor Network (CSTN)	https://www.stjude.org/research/resources-data/childhood-solid-tumor-network/available-resources.html#xenografts	Stewart et al. 2015, 2016
Children’s Oncology Group (COG)	http://www.cogcell.org/xenografts.php	N/A
Pediatric Preclinical Testing Consortium (PPTC)	www.ncipptc.org	N/A
Institut Curie		Boeva et al. 2017

^a^Major sources of reported neuroblastoma PDX/PDOX models as of July 2017

## The tumor microenvironment of neuroblastoma PDOXs

**Fig. 2:**
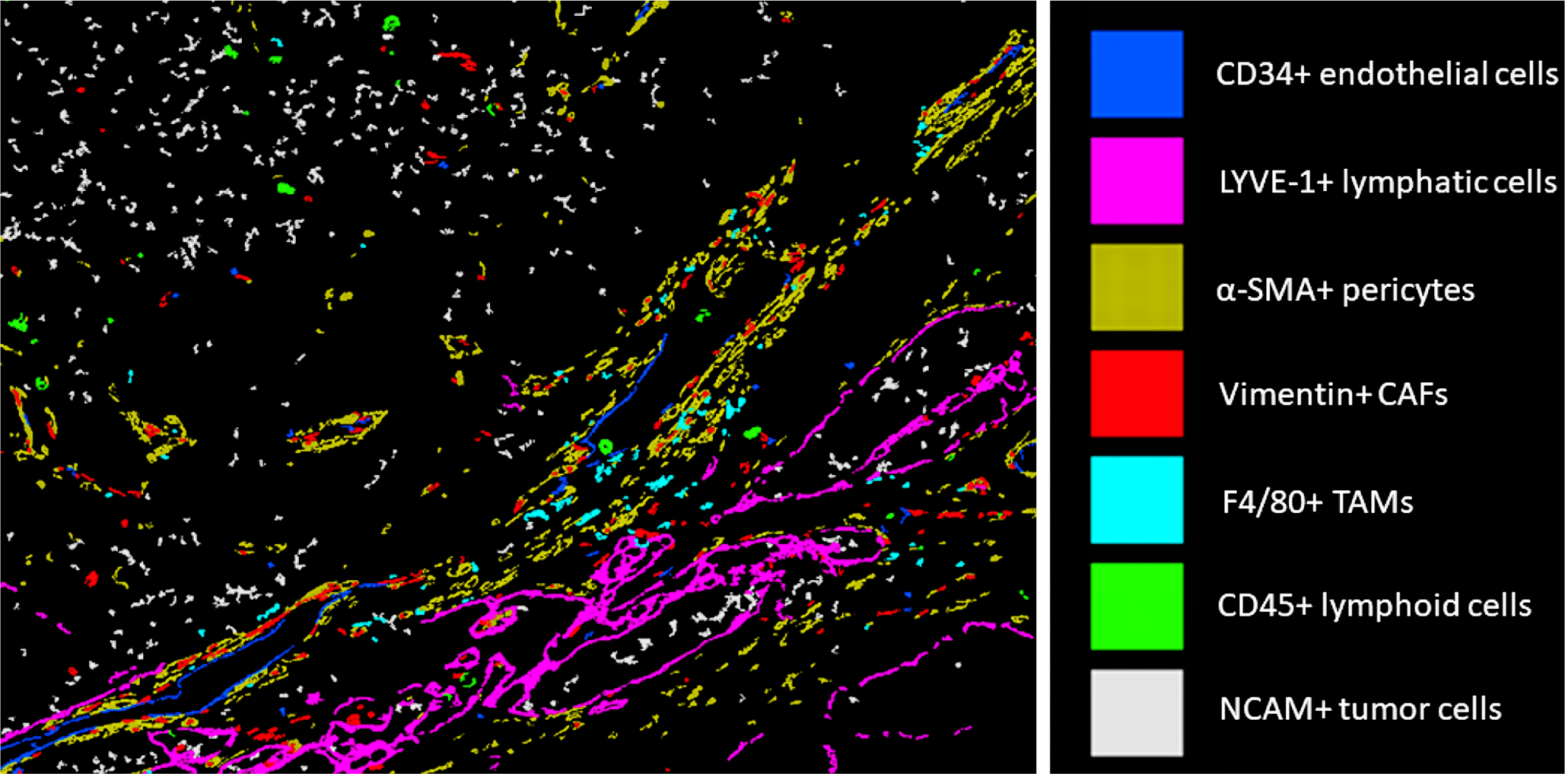
An integrated view of the tumor microenvironment in a neuroblastoma PDOX. Multi-composite image showing the various stromal cell components of a neuroblastoma PDOX grown in an athymic nude mouse. The well-retained structural and cellular complexity is similar to aggressive patient tumors. *CAFs* cancer-associated fibroblasts, *TAMs* tumor-associated macrophages

The tumor microenvironment (TME), or tumor stroma, is critical for cancer progression, metastasis and treatment resistance (Klemm and Joyce 2015). Importantly, neuroblastoma PDOXs contain many critical TME components found in aggressive neuroblastoma patient tumors including tumor-associated macrophages, cancer-associated fibroblasts, pericytes, endothelial cells and extracellular matrix components (Fig. 2) (Braekeveldt et al. 2016). Human endothelial cells derived from the patient’s tumor can be found in a fraction of PDOXs, opening up the possibility of examining and targeting human neuroblastoma–tumor vasculature interactions in vivo. However, mouse endothelium replaces human blood vessels with in vivo passaging, so it is therefore important to establish methods that increase the survival and functional integration of human blood vessels and other human stromal cell types in PDOXs. In addition, the choice of mouse strain affects TME composition. Neuroblastoma PDOXs established in athymic nude mice contain CD45+ lymphocytes and LYVE-1-expressing lymph vessels (Fig. 2), while PDOXs grown in severely immunodeficient NSG mice lack these cell types (Braekeveldt et al. 2016). The finding that many TME components involved in neuroblastoma progression, metastasis and treatment resistance are found in neuroblastoma PDOXs further strengthens their value for preclinical drug testing. However, although there is evidence of human tumor endothelium in some PDOXs, the TME is mainly of murine origin and whether functional crosstalk exists between murine stromal cells and human tumor cells remains to be established.

## Neuroblastoma PDXs in vitro

While conventional cancer cell lines cultured in serum-containing medium as 2D monolayers have their advantages, these cells might not accurately resemble the disease (Gillet et al. 2011; Lee et al. 2006) and only poorly predict clinical outcome (Johnson et al. 2001). 3D cell cultures can, in contrast to 2D monolayers, retain cell–cell and cell–matrix interactions and model hypoxic conditions that better reflect the characteristics of patient tumors. Furthermore, 3D spheroids display increased expression of multidrug resistance genes and survival pathways and are generally more drug resistant than 2D monolayers. 3D spheroids are thus promising in vitro models for drug testing and exploration of treatment resistance (Nath and Devi 2016). We have established free-floating 3D spheroids cultured in serum-free medium from a number of neuroblastoma PDOXs (Braekeveldt et al. 2015). These 3D spheroids retain expression of neuroblastoma markers chromogranin A, synaptophysin and tyrosine hydroxylase and retain their tumorigenic and spontaneous metastatic capacity upon orthotopic injection (Braekeveldt et al. 2015). Similarly, neuroblastoma patient-derived cell lines established in serum-free medium recapitulate the genotype of primary patient tumors (Bate-Eya et al. 2014). As discussed above, patient- or PDX-derived neuroblastoma cell lines have also been established at multiple institutions, including as part of the PPTP, the CSTN program and the COG program.

Organoids are 3D cultures grown on top of or embedded within a matrix such as Matrigel or collagen (Baker et al. 2016). The matrix provides an artificial extracellular matrix niche and prevents cell attachment to the culture dish. Similar to 3D spheroids, organoids are highly promising tools for assessing therapeutic responses as has already been shown for diverse adult tumor types (Baker et al. 2016; Gao et al. 2014; Pauli et al. 2017).

Establishing and handling (passaging, cryopreservation, etc.) tumor cells in serum-free medium requires more experience, patience and time compared to classical serum-cultured cell lines. However, in light of the aberrant changes seen in serum-cultured cells and the preservation of molecular features in cells cultured in 3D in serum-free medium, we believe that this is well worth the effort. Furthermore, 3D-cultured human tumor cells provide a means to implement the important principles of the 3Rs (Replacement, Reduction and Refinement) to minimize animal experimentation.

## Current limitations of PDX models

Despite the promise of PDX models, they also have their limitations: (1) an abnormal immune system in the host mice, (2) the murine tumor microenvironment and (3) tumor heterogeneity. First, athymic nude mice lack functional T cells, NOD-scid mice lack functional T and B cells, while the NOD-scid-gamma (NSG) strain lack functional T, B and NK cells. The immunosuppressed status of these mice precludes immunotherapy testing until methods have been established to reconstitute the human immune system in the animals. It is conceivable that other therapeutic strategies also affect immune cells, a parameter not included in xenograft studies. Attempts are being made to reconstitute the human immune system in mice by injection of human hematopoietic stem cells (Morton et al. 2016) and by other methods (Shultz et al. 2014). However, these approaches are currently expensive and challenging for most laboratories involved in preclinical drug testing.

Second, although the TME of orthotopic PDXs can contain similar cell types as patient neuroblastomas, these cells are mainly of murine origin (Braekeveldt et al. 2016). Functional crosstalk between murine stroma and human tumor cells is not fully understood. Co-injection of human tumor cells with patient-derived stromal cells (e.g., cancer-associated fibroblasts or mesenchymal stem cells) can potentially restore parts of the human TME, as shown in breast cancer PDXs (DeRose et al. 2011).

Third, aggressive tumors (including pediatric cancers) contain various subclones with different genotypes resulting in intratumoral heterogeneity (McGranahan and Swanton 2017; Mengelbier et al. 2015). Furthermore, targetable mutations, possibly driving tumor progression and/or treatment resistance, can be found in subpopulations of aggressive tumors, e.g., *ALK* mutations in neuroblastoma (Schleiermacher et al. 2014). Establishing neuroblastoma PDXs based on the implantation of single tumor biopsies might thus not cover the entirety of the wide genomic changes found in high-risk patient tumors. For instance, PDXs derived from a single sample could lack the mutation(s) driving treatment relapse in the patient. Implantation of multiple samples from relapsed tumors and possibly injection of circulating tumor cells, could potentially solve this issue.

## Application of neuroblastoma PDXs to preclinical drug testing

**Fig. 3:**
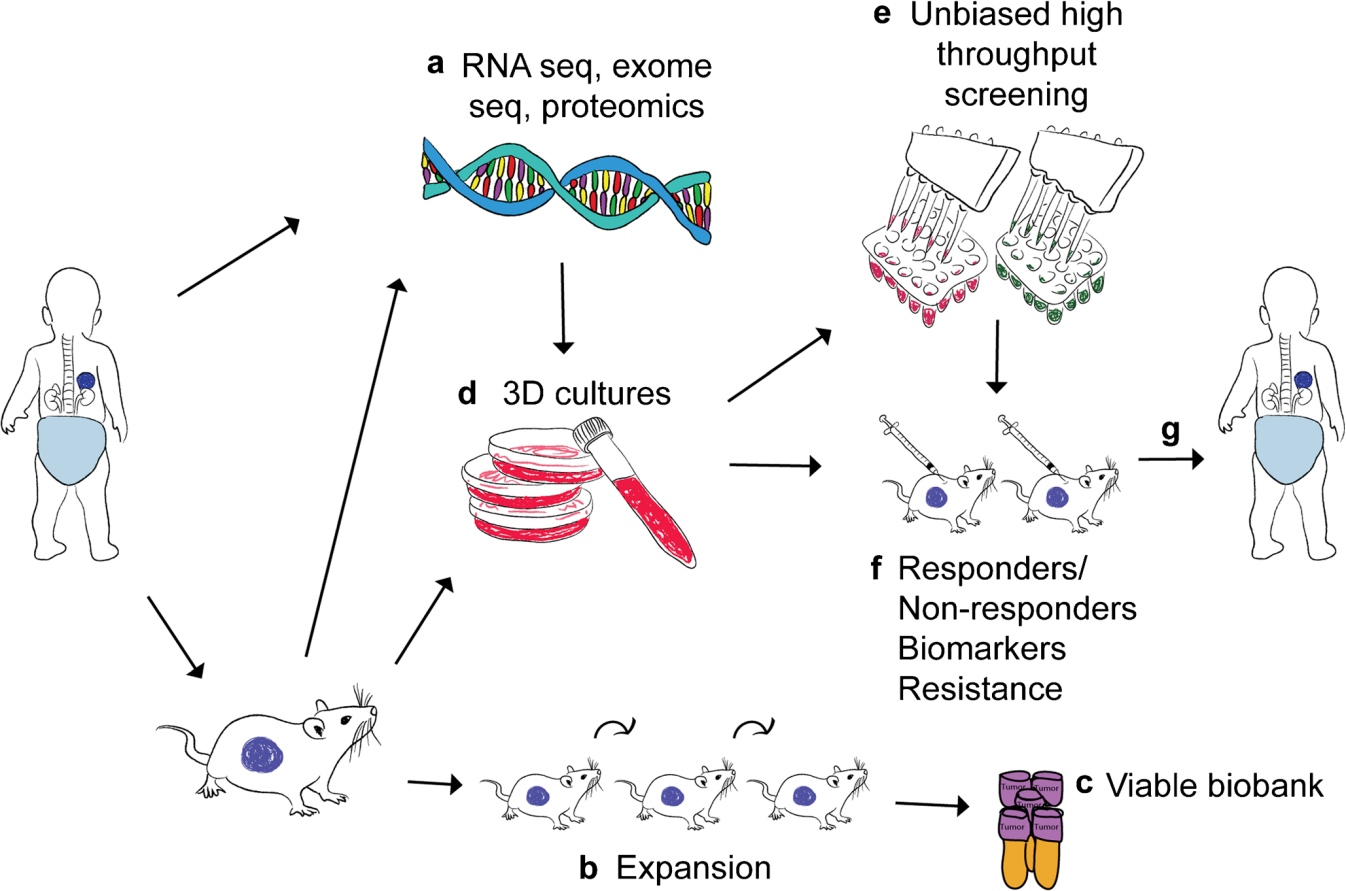
Patient-derived xenografts (PDXs) in translational pediatric cancer research. **a** Both patient tumors and PDXs undergo comprehensive molecular characterization including genomic and proteomic analysis. **b** PDX tumors are passaged in vivo to increase material for drug testing. **c** Viably cryopreserved PDXs or PDX-derived cultured cells can be stored in a biobank for future use. **d** Targeted therapies are selected based on genomic analysis and tested in 3D spheres or organoids. **e** Alternatively, an unbiased high-throughput chemical screen is performed. **f** Promising drug candidates are subsequently tested in multiple PDXs in vivo where responders/non-responders, biomarkers and mechanisms of effect and resistance can be further examined. **g** The results can potentially guide the design of therapy for children with cancer

Despite the limitations of the model system, PDXs have been shown to parallel clinical outcome in various tumor types (Hidalgo et al. 2014; Rosfjord et al. 2014). The major applications of neuroblastoma PDXs/PDOXs relate to drug testing, exploration of treatment resistance and biomarker discovery. The two main approaches to drug testing are targeted therapy based on genomic testing and unbiased high-throughput screening using diverse compound libraries (Fig. 3). In this setting, drug testing of PDXs from multiple patients (instead of, for example, ten mice from one cell line-derived xenograft) can better reflect inter-patient heterogeneity and identify responders/non-responders and biomarkers (Fig. 3). The so-called “co-clinical trial” describes the establishment of a personalized PDX model, a so-called “avatar” model, from a patient enrolled in a clinical trial, with the PDX treated with the same drug(s). Molecular characterization of the patient tumor and PDX can guide genomic-based targeted therapy of the PDX and support clinical decision-making. This strategy could potentially also identify resistance mechanisms and facilitate testing of novel drugs to overcome treatment relapse (Fig. 3). One limitation of the avatar concept is the time frame, since it can take several months to establish a neuroblastoma PDX and the co-clinical trial risks being too slow for real-time clinical decision-making of high-risk patients. Furthermore, pharmacokinetic differences between mice and humans are often not considered, sometimes making direct comparisons difficult.

As described above, the PPTP has undertaken various drug testing protocols against pediatric cancers including neuroblastoma (Houghton et al. 2007; Kang et al. 2011). Several anti-cancer agents have been tested using in vivo and in vitro models and the effects of some of these agents are consistent with their known clinical activity (Houghton et al. 2007; Kang et al. 2011). Recently, neuroblastoma PDXs were utilized to target the PI3K pathway (Chanthery et al. 2012; Mohlin et al. 2015; Stewart et al. 2015) and the *ALK* oncogene (Krytska et al. 2016) for nanofiber-mediated local delivery of the active metabolite of irinotecan (SN-38) (Monterrubio et al. 2016), for delivery of SN-38 nanoparticles conjugated to 3F8 antibodies thereby targeting GD2-expressing cells (Monterrubio et al. 2017) and for concurrent ketoconazole and fenretinide treatment (Lopez-Barcons et al. 2017). However, PDXs have yet to be fully exploited to improve treatment strategies for high-risk neuroblastoma.

The finding that targetable mutations are enriched in relapsed neuroblastomas opens up opportunities for neuroblastoma PDXs in personalized medicine strategies based on genomic testing. Ongoing programs that include comprehensive molecular characterization of relapsed pediatric cancers for individualized targeted therapy include “Pediatric MATCH” (https://www.cancer.gov/about-cancer/treatment/clinical-trials/nci-supported/pediatric-match), MAPPYACTS (https://clinicaltrials.gov/ct2/show/NCT02613962), SMPaeds, iTHER and INFORM (Worst et al. 2016). It is likely that testing single or combination therapies in well-characterized neuroblastoma PDXs will play a crucial role in the development of personalized and precision medicine based on genomic testing. Such initiatives include the NCI-funded Pediatric Preclinical Testing Consortium (http://www.ncipptc.org/), the IMI2 ITCC-P4 program (http://cordis.europa.eu/project/rcn/210764_en.html) and the EU funded Targeting Of Resistance in PEDiatric Oncology program (TORPEDO, http://www.transcanfp7.eu/index.php/abstract/torpedo.html). Genomics analyses of relapsed neuroblastomas indicate that such treatments could include inhibition of ALK and the RAS-MAPK pathway, YAP inhibition and/or pathways involved in EMT (Eleveld et al. 2015; Padovan-Merhar et al. 2016; Schramm et al. 2015).

Although PDX models are useful for preclinical testing, the model is relatively labor-intensive, expensive and low throughput; it therefore lacks the flexibility required for target screening. Unbiased high-throughput chemical screens of thousands of compounds using patient- or PDX-derived cells have been applied to diverse adult tumor types (Crystal et al. 2014; Kitambi et al. 2014; Pauli et al. 2017). This approach can identify candidate drugs not associated with specific mutations but that still exert potent inhibitory effects, as recently shown in glioblastoma (Kitambi et al. 2014). This large-scale approach has also been performed using cultured neuroblastoma patient-derived cells, in which Polo-like kinase 1 was identified as a therapeutic target (Grinshtein et al. 2011). In a pilot study using high-throughput screening of short-term cultured neuroblastoma PDOX-derived cells, the cells were found to be much less sensitive to chemotherapy than conventional neuroblastoma cell lines (Stewart et al. 2015).

A number of important and sometimes overlooked issues and parameters exist in pediatric PDX preclinical drug testing studies that must be considered to increase their clinical relevance and predictive value. These include using clinically relevant drug doses, consideration of pharmacokinetics and pharmacodynamics, use of relevant drug combinations and treatment schedules and measuring toxicity. Furthermore, the methods used to measure success in preclinical studies often differ from clinical studies. Effects measured by differences in tumor volume or survival between treatment groups are often defined as successes in preclinical studies. However, although statistically significant differences between treatment groups exist, treated xenografts often progress and similar effects seen in the clinical setting would be regarded as progressive disease and failure. Comprehensive recommendations to increase the clinical relevance of preclinical studies of pediatric cancer are outlined in another review (Langenau et al. 2015).

## Methodological issues

There are a number of methodological issues regarding the establishment and use of PDXs as preclinical neuroblastoma models. Here, we discuss eight important issues that researchers using or intending to use these models might wish to consider.

1. Patient information and informed written consent are critical for the establishment of PDX models.
2. Neuroblastoma is a rare tumor, and many academic centers do not have access to enough samples. Cryopreservation of fresh tumor fragments/cells can solve this issue. Briefly, fresh tumor fragments (approximately 1–2 × 1–2 mm) are stored in ice-cold medium and cryopreserved through stepwise cooling in cryopreservation medium. These cryopreserved and viable samples can be stored and/or shipped on dry ice to another laboratory for implantation. We have previously shown that it is feasible to establish PDOXs from cryopreserved high-risk neuroblastoma samples (Braekeveldt et al. 2015). Findings from other tumor types suggest that the engraftment rates of fresh and cryopreserved tumor material are similar (Linnebacher et al. 2010; Sorio et al. 2001). In this way, laboratories without direct access to neuroblastoma patient material can, through collaborations, establish PDXs.
3. Clearly, establishing additional neuroblastoma PDXs from relapsed or post-mortem tumors would be very useful for testing therapies against treatment-resistant disease. Collection and injection of circulating tumor cells can also add value to the PDX model system (Girotti et al. 2016). It is also important to increase the pool of neuroblastoma PDXs from not only relapsed but also metastatic sites.
4. Non-dissociated tumor fragments are commonly utilized to establish and serially propagate PDXs. As discussed above, cell culturing can lead to molecular and phenotypic deviations from the original patient tumor (Baysan et al. 2014; Lee et al. 2006). However, for mechanistic and treatment studies, it is often necessary to inject dissociated cultured cells. If possible, short-term cell cultures grown under serum-free conditions should be applied.
5. There is a possibility of contamination of tumor cells. First, patient tumor cell cultures can easily be contaminated with EBV-infected B lymphoblasts from the same patient tumor sample. Unfortunately, proliferative EBV lymphoblasts can, after prolonged culturing or in vivo growth, completely overgrow the tumor cells (Bondarenko et al. 2015; Dieter et al. 2017; Mohlin et al. 2012). Regular genotypic confirmation of PDX tumor cells is thus recommended. Second, cross-contamination of tumor cells is a well-known problem in cancer research, necessitating authentication testing (Capes-Davis et al. 2010).
6. The site of implantation/injection will affect the features of established tumors. In experiments comparing subcutaneous injection of neuroblastoma cells versus orthotopic injection into the adrenal gland, orthotopic tumors retained a more relevant biological phenotype and spontaneous metastases to distant organs (Khanna et al. 2002). Indeed, findings from many other tumor types have shown that orthotopic PDXs retain higher metastatic capacity compared to subcutaneous PDXs (Hoffman 2015). Thus, it seems that neuroblastoma PDOXs are required to study and target neuroblastoma metastasis. However, establishing and monitoring neuroblastoma orthotopic tumors is time consuming and requires in vivo imaging methods such as magnetic resonance imaging (Braekeveldt et al. 2015).
7. Not all patient tumor samples engraft in mice. The tumor engraftment rate using orthotopic implantations is around 50% (Braekeveldt et al. 2015). However, our experience and that of others suggest that the tumor engraftment rate can be much lower with subcutaneous implantation. Our experience is that high-risk neuroblastomas are easier to engraft, whereas PDXs from low-risk patient tumors are much more difficult to establish. Potential approaches to increase the engraftment rate include the use of Matrigel and/or co-injection with supporting stromal cells (DeRose et al. 2011).
8. A final issue relates to the choice of mouse strain. Traditionally, patient samples have been implanted into athymic nude mice or scid mice to establish patient xenografts. We and others utilized severely immunodeficient NSG mice lacking T, B and NK cells, based on the assumption that these mice are more permissive to tumor engraftment due to their immunosuppressed status. Indeed, injection of patient-derived malignant melanoma cells results in much higher engraftment in NSG mice compared to NOD-scid mice (Quintana et al. 2008). However, a direct comparison of neuroblastoma engraftment rate between nude, NOD-scid and NSG mice has, to our knowledge, not been performed. As discussed above, the choice of mouse strain can also affect the TME of established PDXs (Braekeveldt et al. 2016).

## Conclusions

Subcutaneous and orthotopic neuroblastoma PDXs have been established at various institutions internationally and these PDXs retain the molecular and phenotypic features of patient tumors. Furthermore, neuroblastoma PDOXs retain robust spontaneous metastatic capacity and are therefore excellent models for targeting neuroblastoma metastasis. PDXs from various tumor types can predict clinical outcome, making them extremely good models for biomarker and preclinical drug development. Neuroblastoma PDXs might thus play an important role for personalized and precision medicine strategies against treatment-resistant disease, especially given our recent understanding of the genomic changes associated with relapsed neuroblastomas. However, establishing and characterizing additional PDXs from high-risk and relapsed tumors is crucial to cover the different biological subsets of high-risk neuroblastoma. Furthermore, initiation of a global clinical academic collaboration to include and share all existing neuroblastoma PDXs would increase the utility of these promising models. Despite their limitations, neuroblastoma PDXs hold promise to improve the treatment of children with high-risk metastatic neuroblastoma.
